# Supplementary material for: Cassini spacecraft reveals global energy imbalance of Saturn
Source: Nat Commun. 2024 Jun 18;15:5045. doi: 10.1038/s41467-024-48969-9 (PMC11189510; doi:10.1038/s41467-024-48969-9)
Supplement: Supplementary file 1 — Supplementary Information [file 41467_2024_48969_MOESM1_ESM.pdf]

Supplementary Information for

Cassini Spacecraft Reveals Global Energy Imbalance of Saturn

Xinyue Wang[1], Liming Li[2*], Xun Jiang[1], Patrick M. Fry[3], Robert A. West[4], Conor A. Nixon[5], Larry Guan[2], Thishan D. Karandana G.[1], Ronald Albright[1], Joshua E. Colwell[6], Tristan Guillot[7], Mark D. Hofstadter[4], Matthew E. Kenyon[4], Anthony Mallama[8], Santiago Perez-Hoyos[9], Agustin Sanchez-Lavega[9], Amy A. Simon[5], Daniel Wenkert[4], Xi Zhang[10]

[1] *Department of Earth and Atmospheric Sciences, University of Houston, Houston, TX, USA, 77004.*
[2] *Department of Physics, University of Houston, Houston, TX, USA, 77004.*
[3] *Space Science and Engineering Center, University of Wisconsin-Madison, Madison, WI, USA, 53706.*
[4] *Jet Propulsion Laboratory, California Institute of Technology, Pasadena, CA, USA, 91109.*
[5] *NASA Goddard Space Flight Center, Greenbelt, MD, USA, 20771.*
[6] *Department of Physics, University of Central Florida, Orlando, FL, USA, 32816.*
[7] *Université Côte d’Azur, Observatoire de la Côte d’Azur, CNRS, Laboratoire Lagrange, Nice, France, 06108.*
[8] Department of Mathematics and Statistics, University of Maryland, College Park, MD, USA, 20742.
[9] *Departamento de Fisica Aplicada I, Escuela de Ingenieria UPV/EHU, Bilbao, Spain, 18013.*
[10] *Department of Earth and Planetary Sciences, UCSC, Santa Cruz, CA, USA, 95064*
[*] To whom all correspondence should be addressed. E-mail: lli7@central.uh.edu

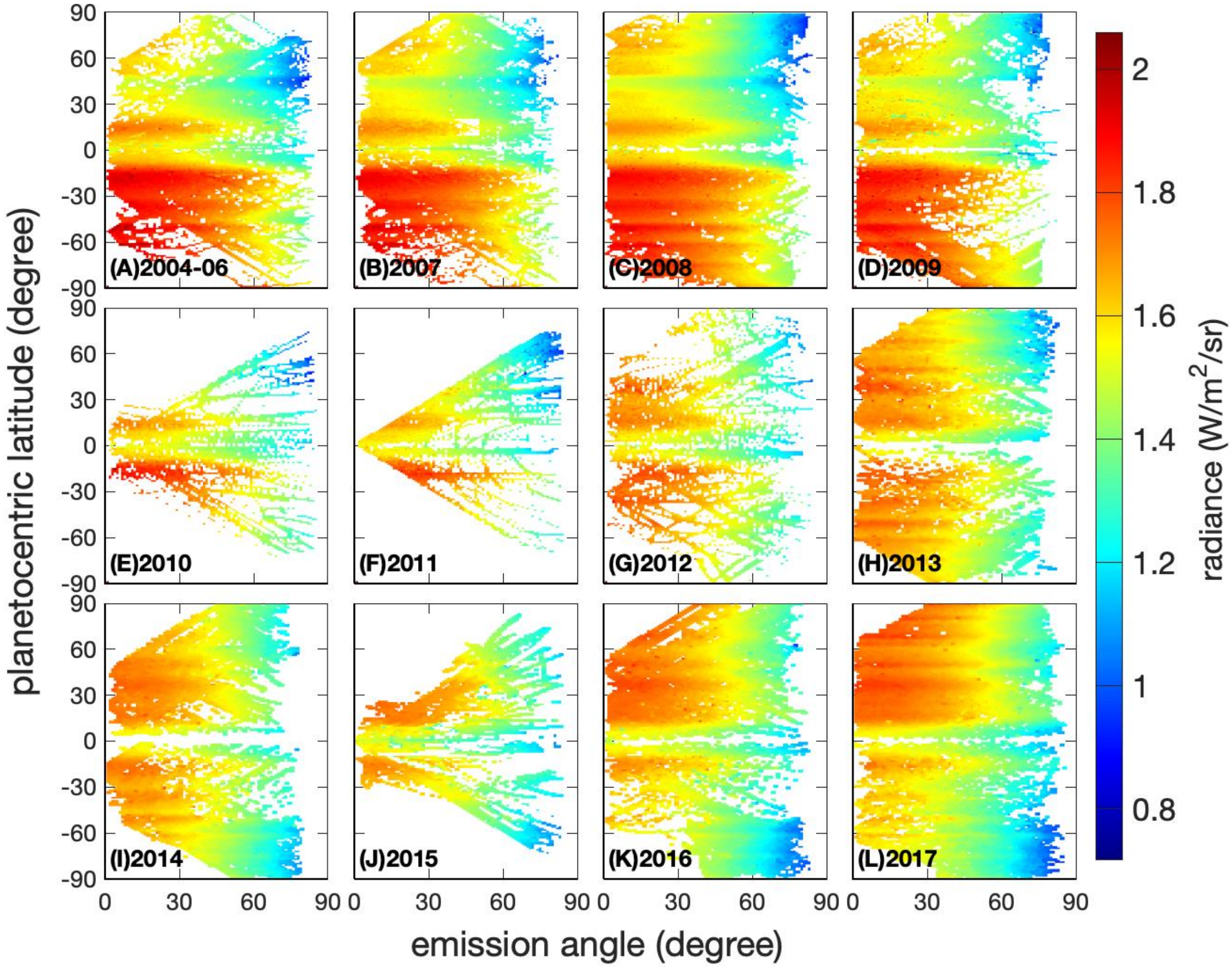

Figure S1. Coverage of Cassini/CIRS observations. The wavenumber-integrated radiance is shown in a two-dimensional domain of emission angle and latitude for different Earth years. The CIRS observations in 2004 and 2005 are relatively sparse, so we combined them with the observations in 2006. Only the radiance recorded by the first focal plane (FP1), which is dominant in Saturn's total emitted power, is displayed. Observations by FP3 and FP4, which have better coverage in the domain of latitude and emission angle, are not shown.

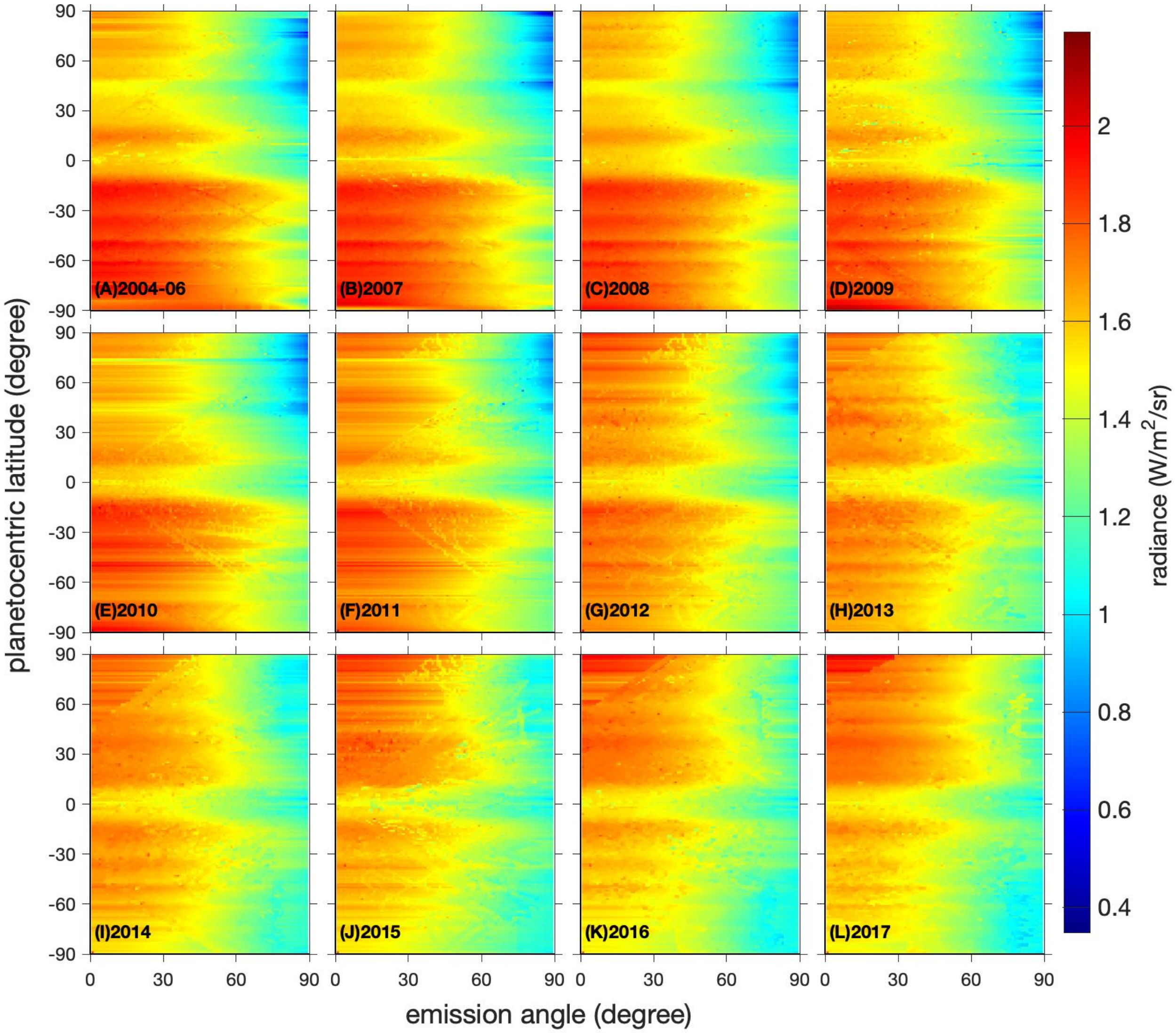

Figure S2. Same as Fig. S1 except for observational gaps filled using linear interpolation/extrapolation in space and time by the least-squares technique.

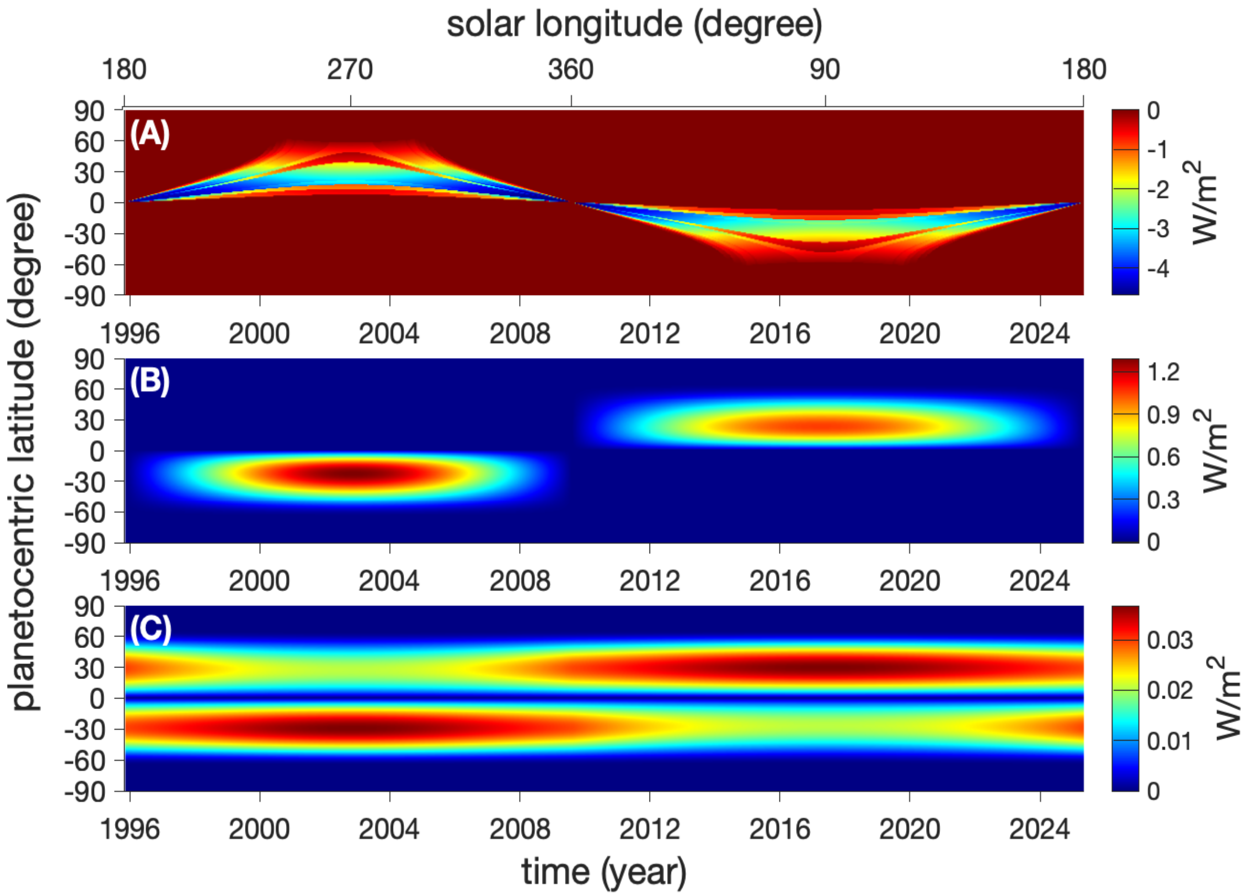

Figure S3. Three effects of the rings on the radiant energy budget of Saturn's atmosphere. (A) Ring-shadowing effect. Saturn's rings cast shadows on the atmosphere, reducing the amount of solar irradiance that reaches the top of the atmosphere. (B) Ring-scattering effect. Saturn's rings scatter some of the incoming solar radiation, enhancing solar irradiance in some latitudes. (C) Ring-emitting effect. The rings also emit thermal radiation, which contributes to the atmospheric energy budget. The time is for a complete orbital period from November 1995 to May 2025, which includes the Cassini epoch (2004-2017). The solar longitude, which corresponds to the time on the bottom, is shown on the top of the figure.

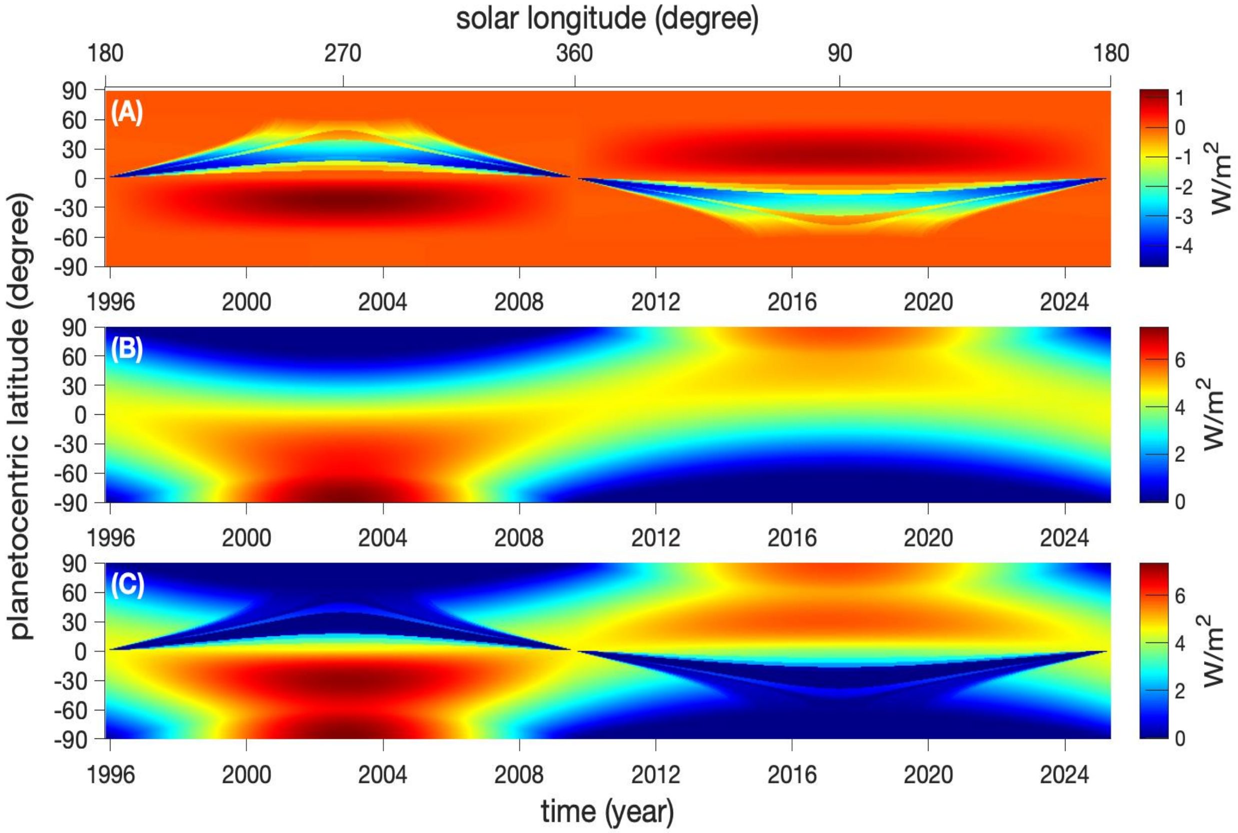

Figure S4. Incident solar irradiance at the top of Saturn's atmosphere. (A) The combination of ring-shadowing and ring-scattering effects. (B) The incident solar irradiance without considering the effects from the rings. (C) The incident solar irradiance including the two effects of the rings (ring-shadowing and ring-scattering).

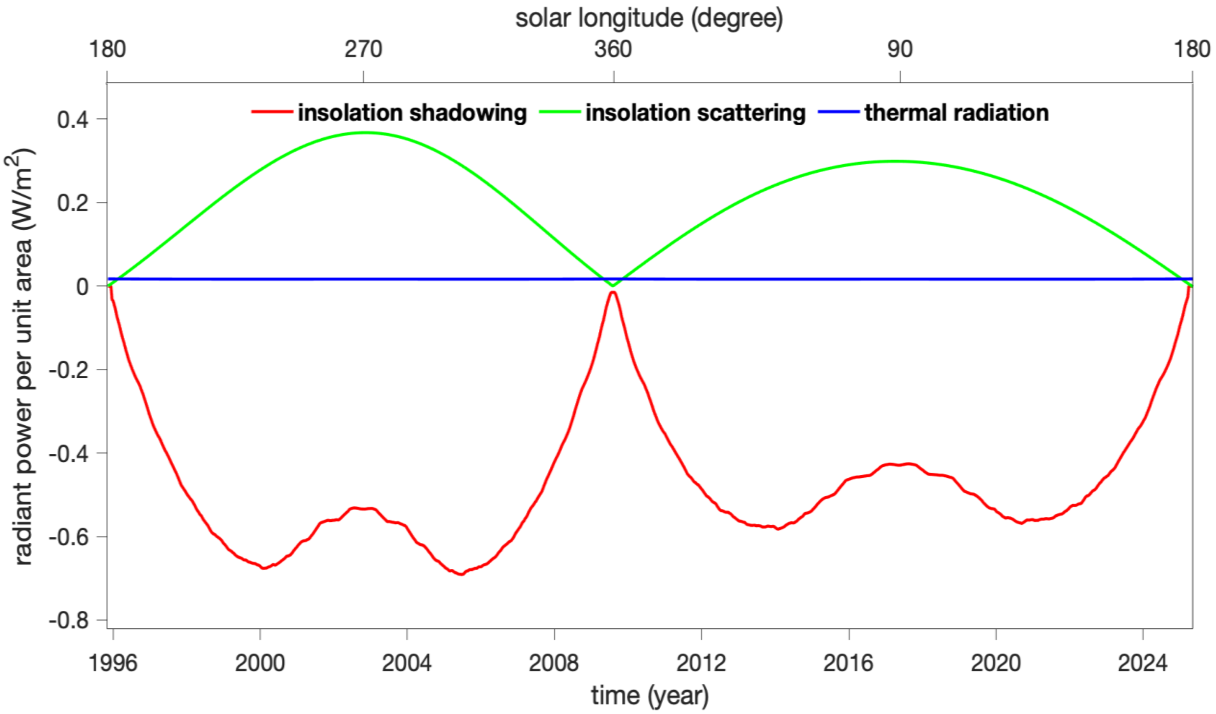

Figure S5. Global averages of three effects of the rings (ring-shadowing, ring-scattering, and ring-emitting). This figure is based on Fig. S3.

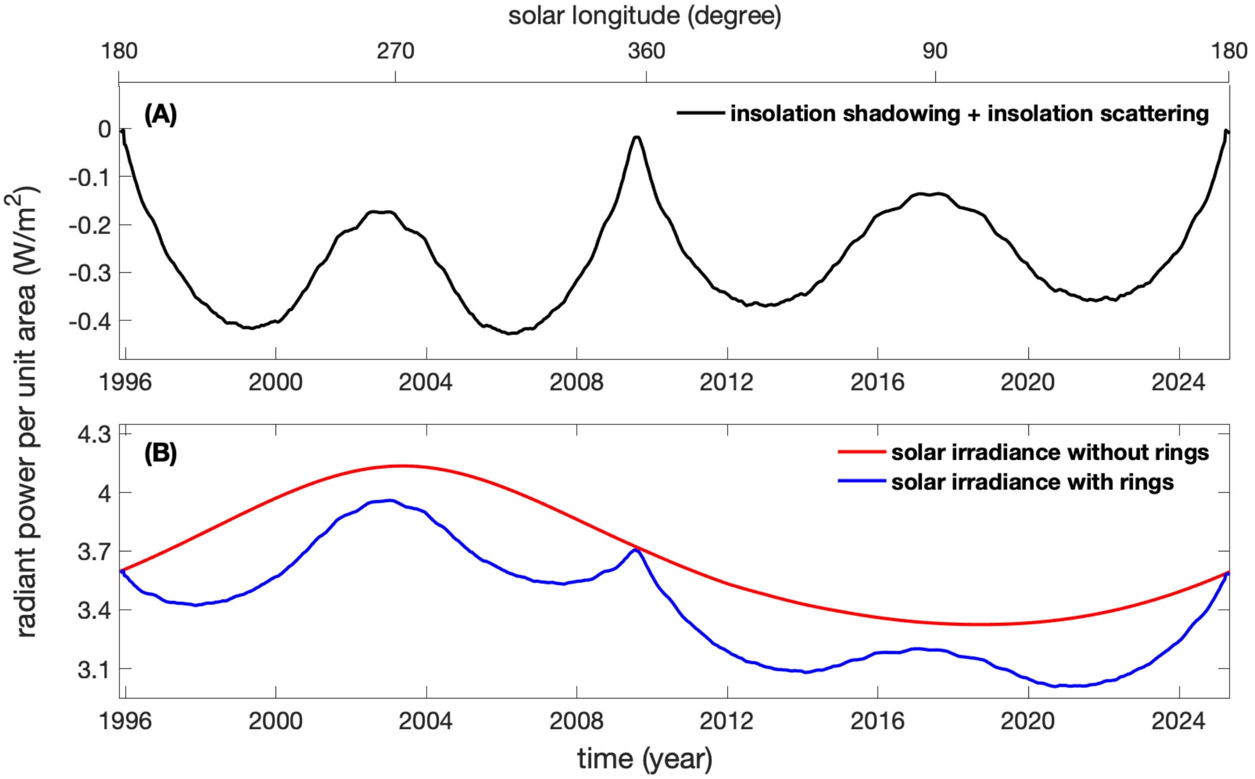

Figure S6. Global averages of the incident solar irradiance at the top of Saturn's atmosphere with and without the effects of the rings. (A) The global average of the combination of the ring-shadowing and ring-scattering effects. (B) The global averages of solar irradiance with and without the effects of the rings.

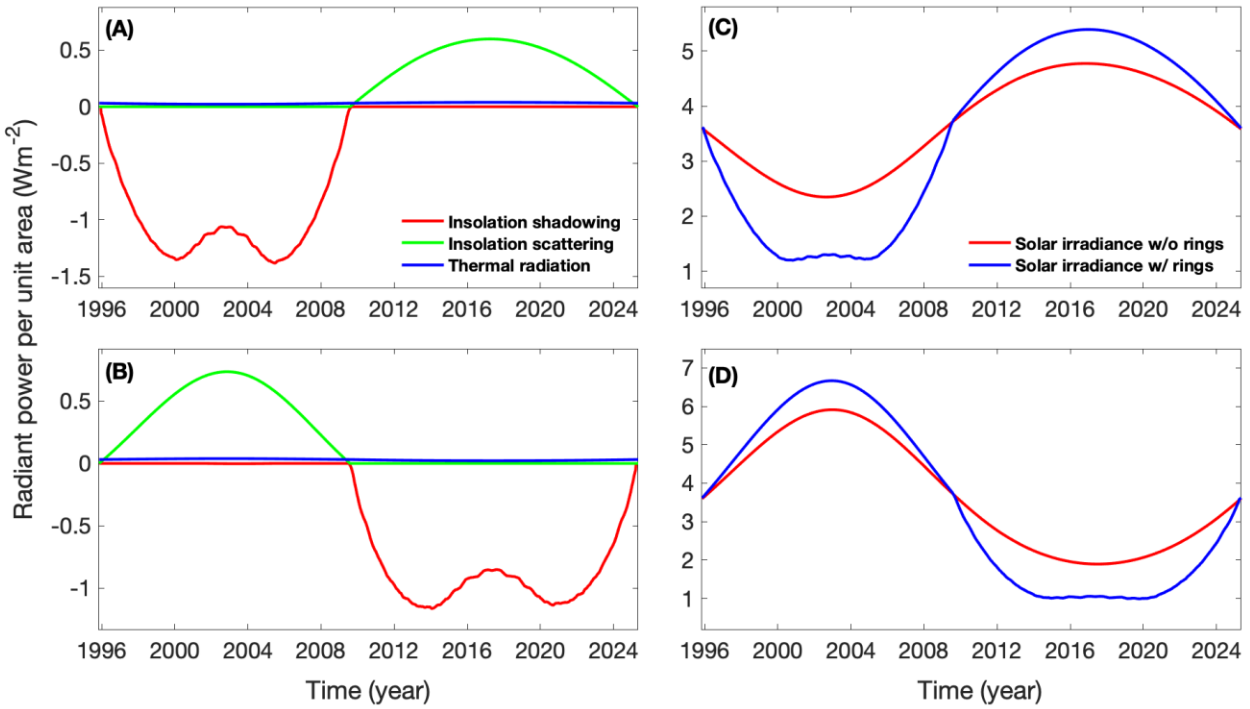

Figure S7. Hemispheric averages of three effects of the rings (ring-shadowing, ring-scattering, and ring-emitting) and solar irradiance with and without the effects of the rings. (A) Three effects of the rings in the NH. Panel (B) is the same as panel (A) except for the analysis of the SH. (C) NH-average of incident solar irradiance at the top of Saturn's atmosphere with and without the effects of the rings. Panel (D) is the same as panel (C) except for the analysis of the SH.

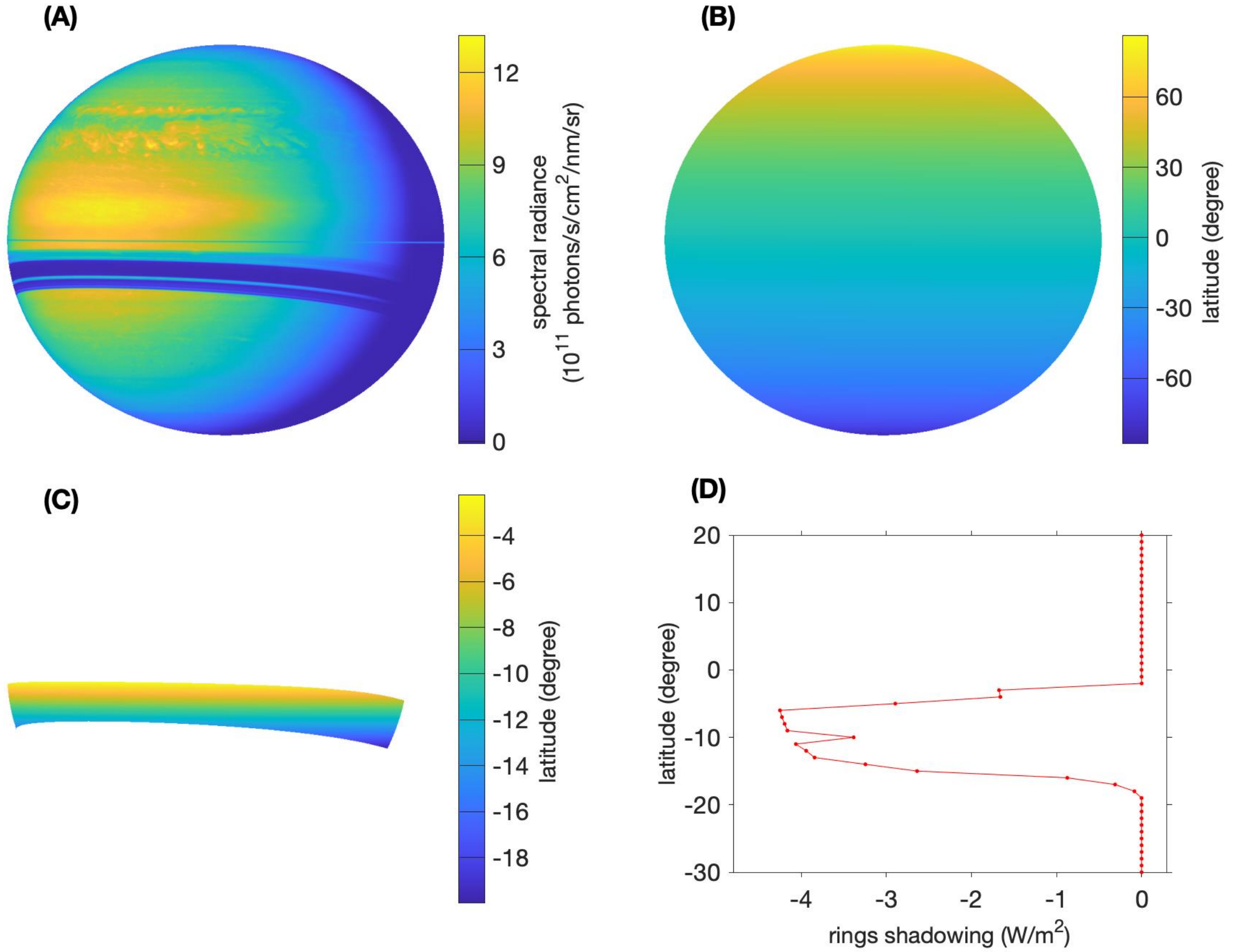

Figure S8. Comparison of the ring-shadowing effect between observations and model simulations. (A) A calibrated image based on a raw image recorded by the Cassini ISS. The raw image (image identification number W1684259810) was recorded by the ISS at the CB2 filter (752 nm) in May 2011 with a phase angle of 35.4º and a spatial resolution of 151 km/pixel. The sub-Cassini-latitude is very small (0.2º), so the rings only block a narrow latitude band around the equator from Cassini's view (see the thin line around the equator in panel A). The observational time (May 2011) corresponds to the spring of the Northern Hemisphere, with a sub-solar-latitude of 9.5ºN, resulting in the shadows cast by the rings appearing in the Southern Hemisphere. (B) Navigated latitude plane for the image shown in panel A. Panel (C) is the same as panel (B) but only the latitudes affected by the rings' shadows are shown. (D) The latitude distribution of the ring-shadowing effect. Such a distribution is outputted from the rings' model for the ISS observational time (May 16, 2011).

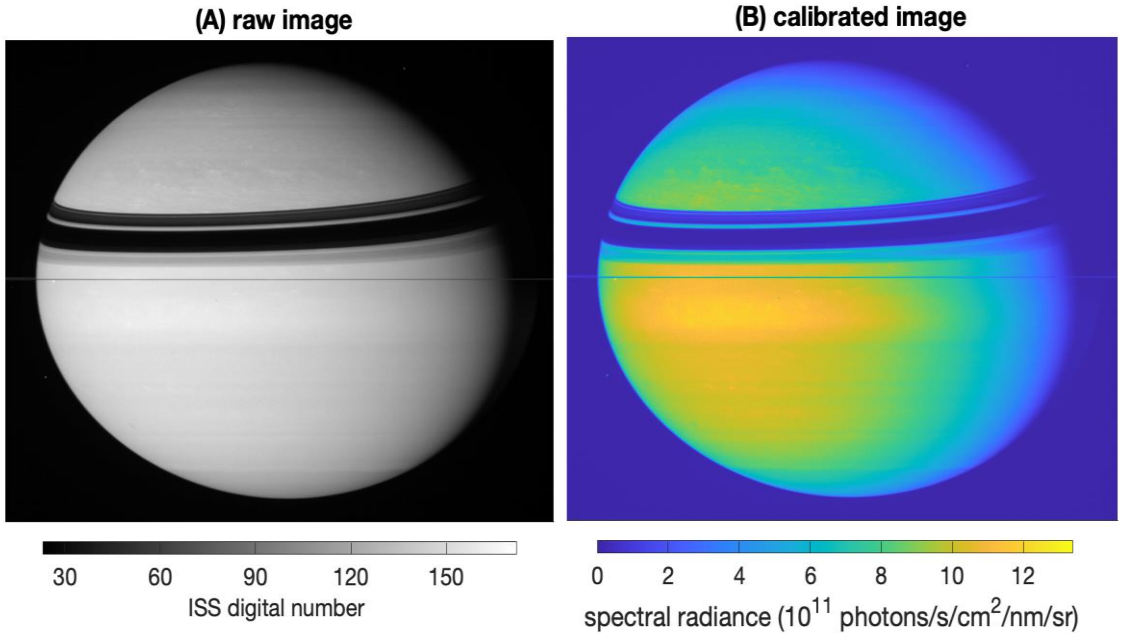

Figure S9. Examples of Cassini/ISS raw and calibrated images. (A) The ISS raw image. (B) The calibrated image with units of spectral radiance. The raw image (image identification number W1564337403) was captured by the ISS RED filter (647 nm) in July 2007 at a phase angle of 30.5° and a spatial resolution of 180.2 km/pixel. The ISS raw images are calibrated using the Cassini ISS CALibration (CISSCAL) software (version 4.3).

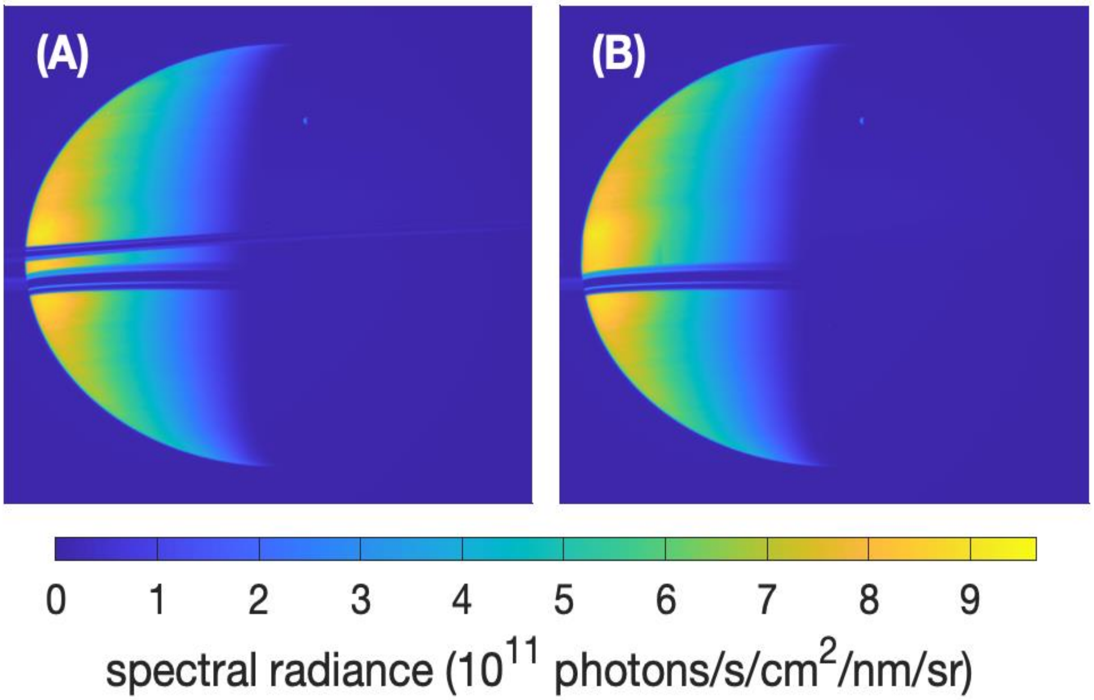

Figure S10. An example of selected ISS images with small sub-Cassini latitudes (< 3º). (A) The raw ISS image. (B) The image after filling in the observational gaps caused by Cassini's view being blocked by the rings. The raw image (image identification number W1657882472) was taken in July 2010 by the ISS CB3 filter (939 nm) at a phase angle of 98.1º and a spatial resolution of 153.2 km/pixel. The sub-Cassini latitude is 2.6ºS, and the sub-solar latitude is 5.1ºN.

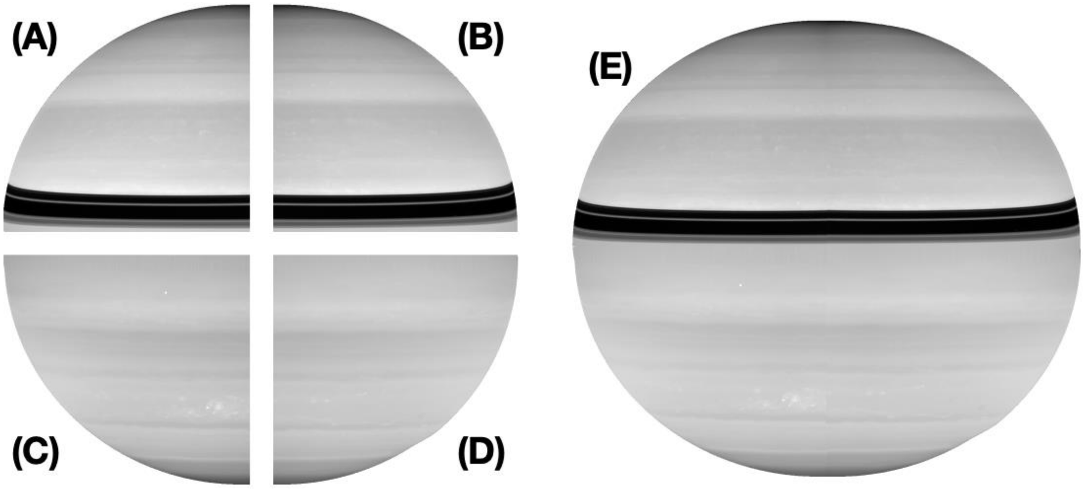

Figure S11. Example of creating global images using ISS quarter images. Panels A, B, C, and D show the four quarter images (image identification numbers are W1591278931, W1591281170, W1591282531, and W1591284770, respectively) taken by the ISS BL1 filter (463 nm) in June 2008 at a phase angle of 15.9° and a spatial resolution of 68 km/pixel. These images were taken under similar viewing geometry in quasi-simultaneous mode, with a time separation of 40 minutes. Therefore, they can be combined to create a global image, as shown in panel E.

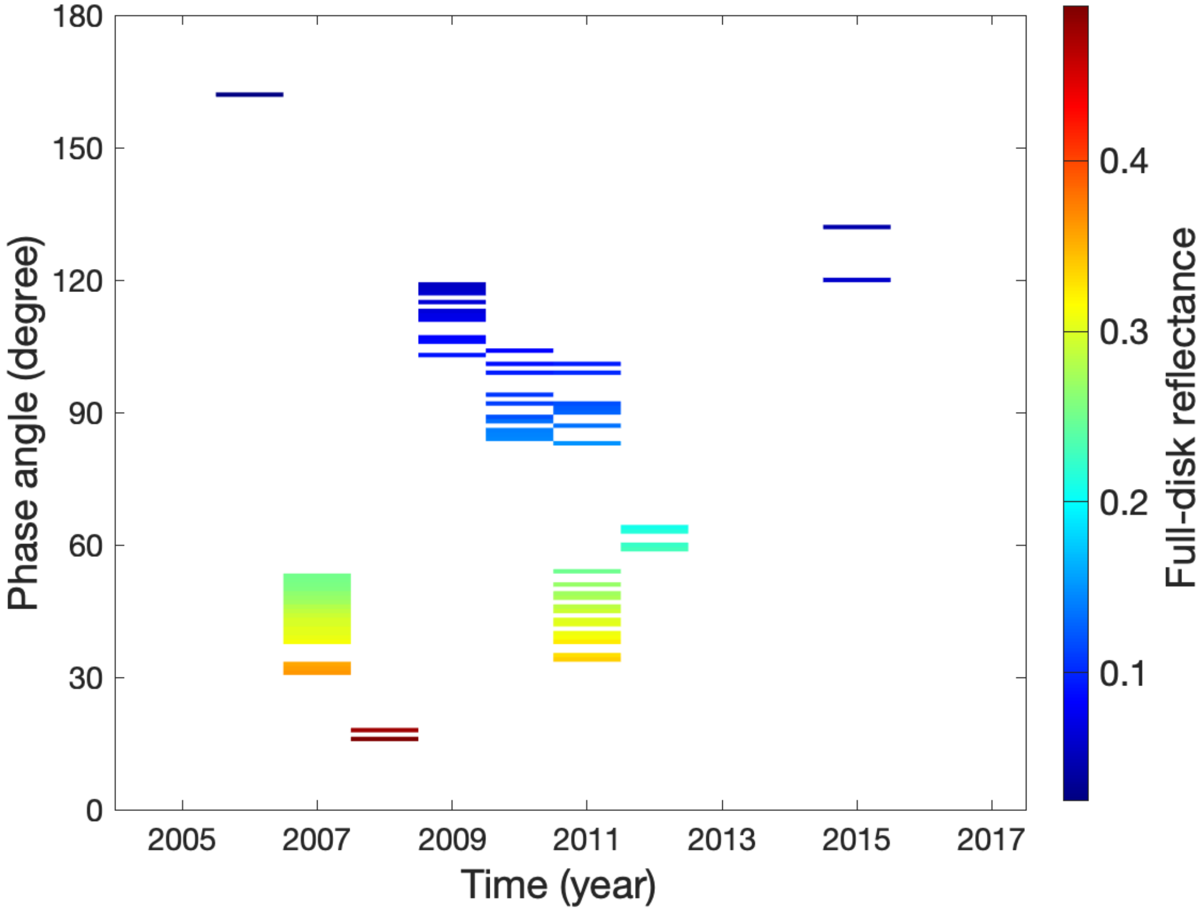

Figure S12. Temporal variability of phase angle coverage for the Cassini/ISS selected global observations. The Cassini/ISS observations recorded at the RED filter (647 nm) are shown in this figure.

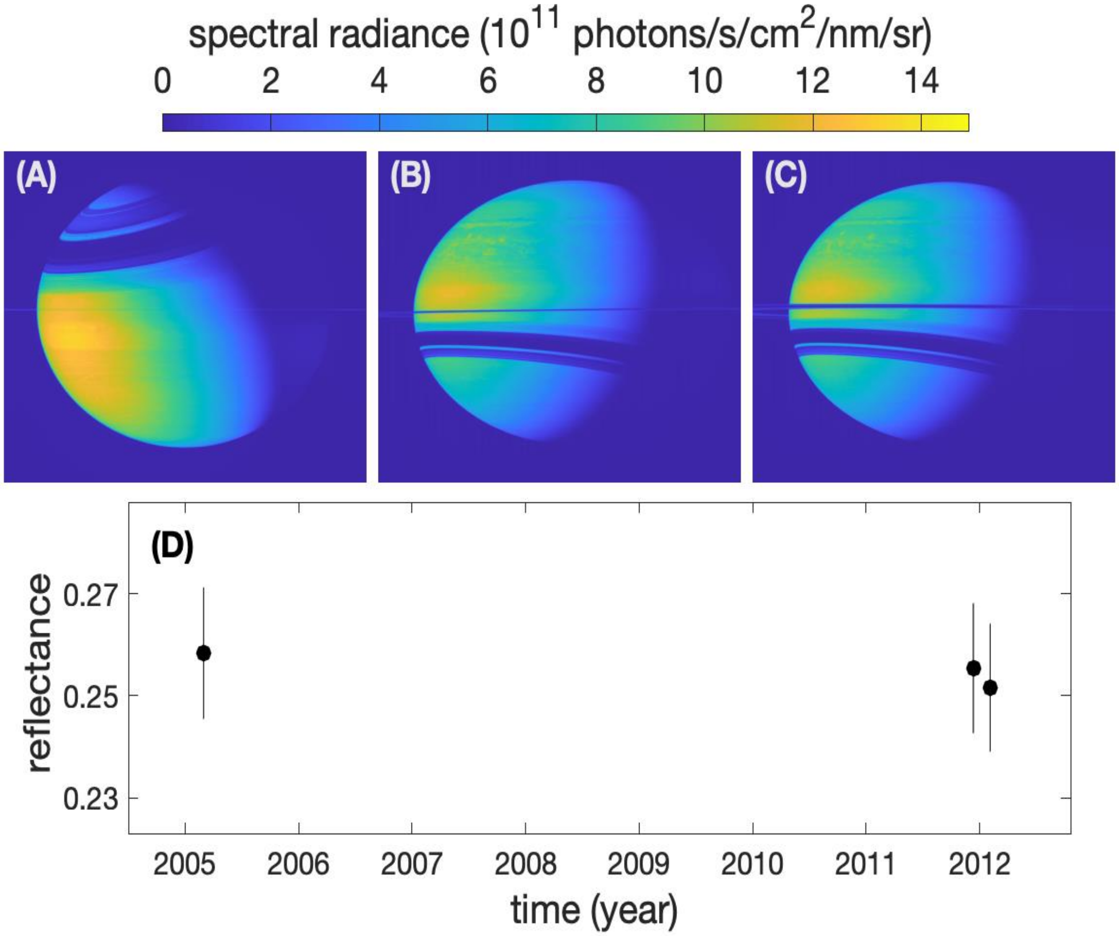

Figure S13. Saturn's full-disk reflectance during the Cassini epoch recorded by the ISS CB2 filter. Panels A-C show the ISS calibrated images recorded by the CB2 filter (752 nm) at three different times but with the same phase angle of 62°. (A) An image (image identification number W1488286907) taken on February 28, 2005 with a spatial resolution of 154.2 km/pixel. (B) An image (image identification number W1703506791) taken on December 25, 2011 with a spatial resolution of 167.5 km/pixel. (C) An image (image identification number W1705630917) taken on January 19, 2012 with a spatial resolution of 168.7 km/pixel. Panel D shows Saturn's full-disk reflectance for the three images shown in panels A-C.

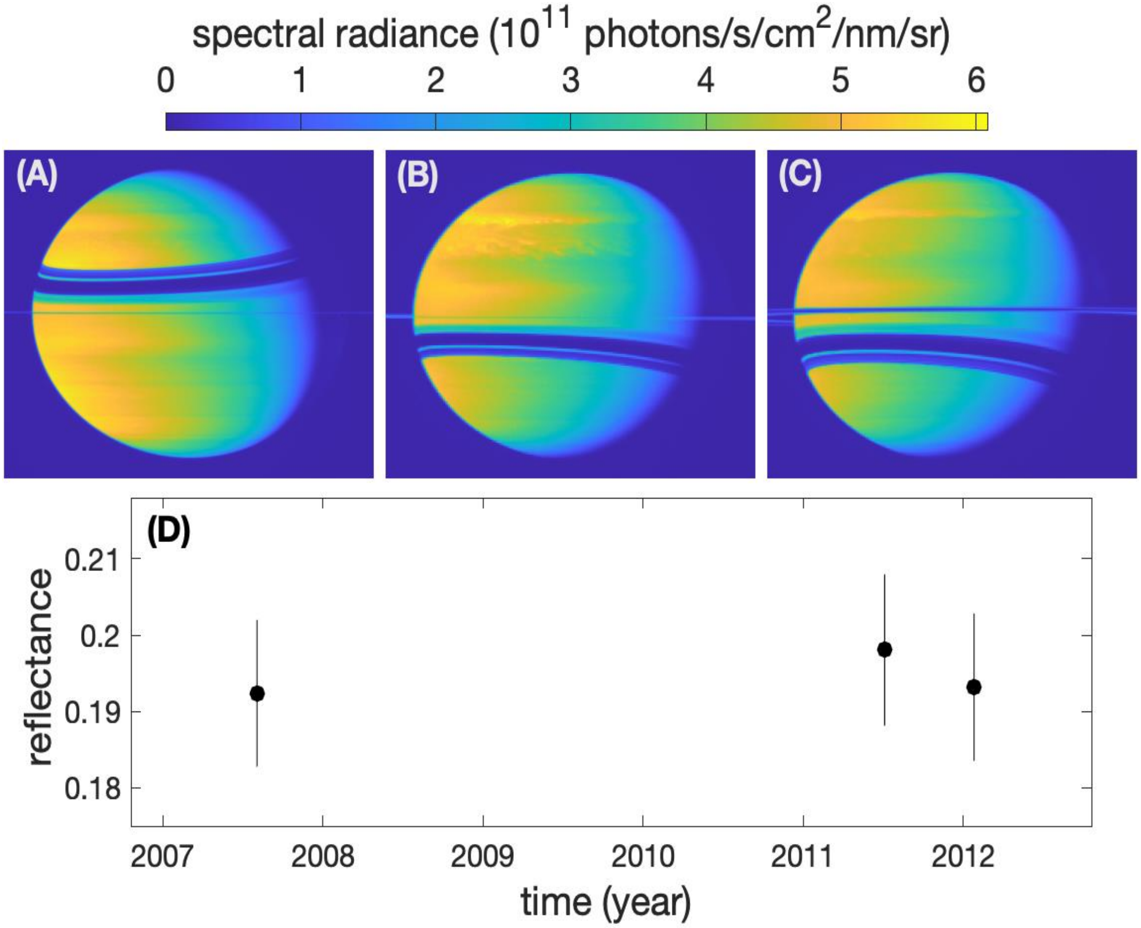

Figure S14. Saturn's full-disk reflectance during the Cassini epoch recorded by the ISS BL1 filter. Panels A-C show calibrated images taken by the BL1 filter at three different times but with the same phase angle of 39°. Panel A shows an image (image identification number W1564774514) taken on August 2, 2007 with a spatial resolution of 224.8 km/pixel. Panel B shows an image (image identification number W1689763636) taken on July 19, 2011 with a spatial resolution of 627.5 km/pixel. Panel C shows an image (image identification number W1704871311) taken on January 10, 2012 with a spatial resolution of 554.1 km/pixel. Panel D shows Saturn's full-disk reflectance for the three images shown in Panels A-C. Note that panel B captured the bright clouds in the middle latitudes of the NH, which were generated by the 2010 giant storm.

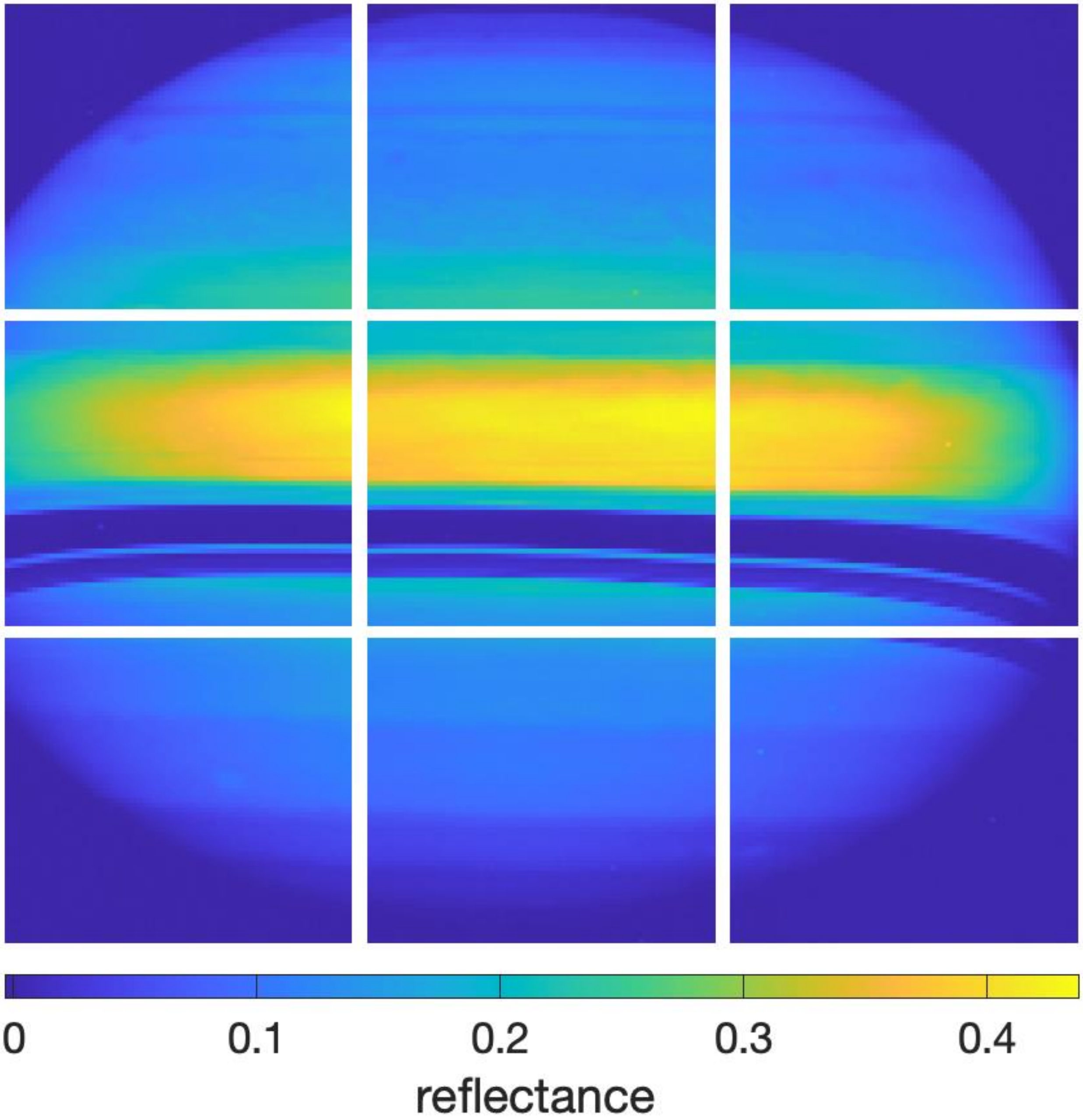

Figure S15. A VIMS global image created from multiple images. The nine VIMS images (image identification numbers are 1689175204, 1689175824, 1689176404, 1689177004, 1689177624, 1689178244, 1689178824, 1689179444, and 1689180004, respectively) used to create the global image were taken by the Cassini VIMS on July 12, 2011, with a phase angle of 11.5° and a spatial resolution of 600 km/pixel. The images were taken under nearly identical viewing geometries using a quasi-simultaneous mode, with a time separation of approximately 10 minutes between neighboring images, allowing them to be processed into a global image. The VIMS has a spectral range of 350-5131 nm with varying spectral resolutions, and the nine images shown here were taken at a wavelength of 2000 nm (2 μm). The VIMS data were calibrated using the Geological Survey ISIS3.

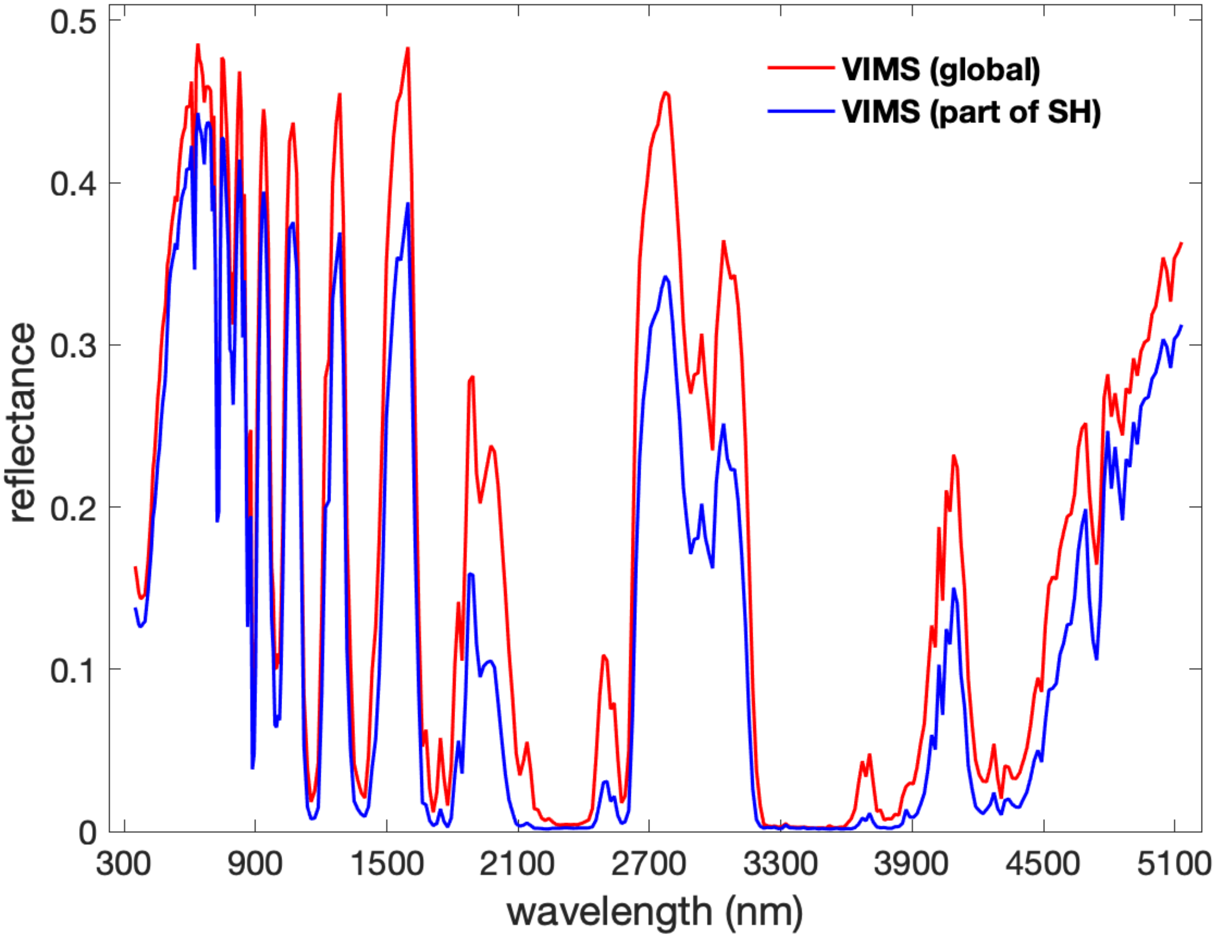

Figure S16. Saturn's Reflectance Spectra Based on VIMS Observations. The red line represents the global spectra, which is a global average based on the quasi-global image shown in Fig. S15, composed of nine VIMS images. The blue line represents the spectra of Saturn's regional areas, based on the middle panel of the bottom three images shown in Fig. S15.

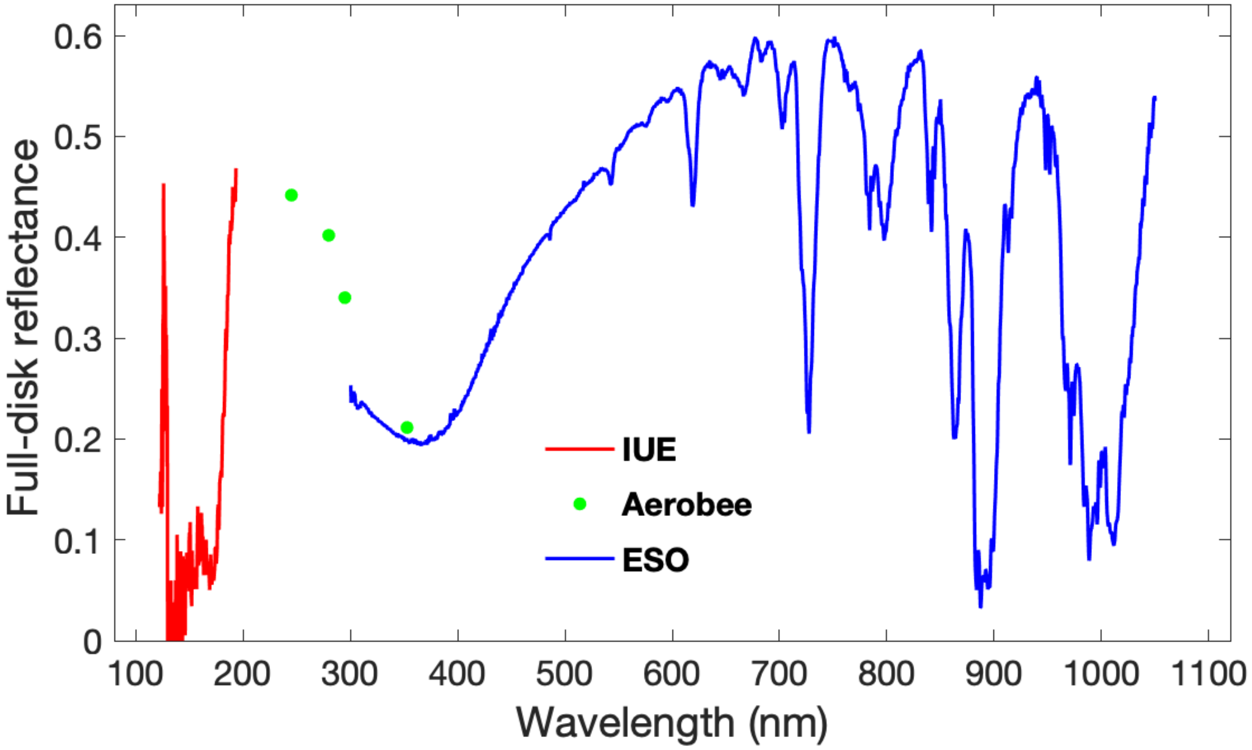

Figure S17. Saturn's full-disk reflectance spectra as observed by observatories other than Cassini. The data recorded by the International Ultraviolet Explorer (IUE) are from a study by Clarke et al[51]. The IUE observed Saturn in 1978-80 with a phase angle close to 0°, covering the spectral range of 120-194 nm with varying spectral resolutions from 0.1 nm to 1.2 nm. The data recorded by Aerobee rocket come from a study by Bless et al[52]. The Aerobee Rocket recorded Saturn's full-disk reflectance in 1964 with a phase angle of 0.89° across four wavelengths from ultraviolet to visible (i.e., 245 nm, 280 nm, 295 nm, and 353 nm). The data recorded by the European Southern Observatory (ESO) come from a previous analysis by Karkoschka[45]. For the ESO data, the Boller and Chivens spectrograph, mounted on a 1.52-m telescope, observed Saturn in 1995 at a phase angle of 5.7°, covering a spectral range of 300-1050 nm with a spectral resolution of 0.4 nm.

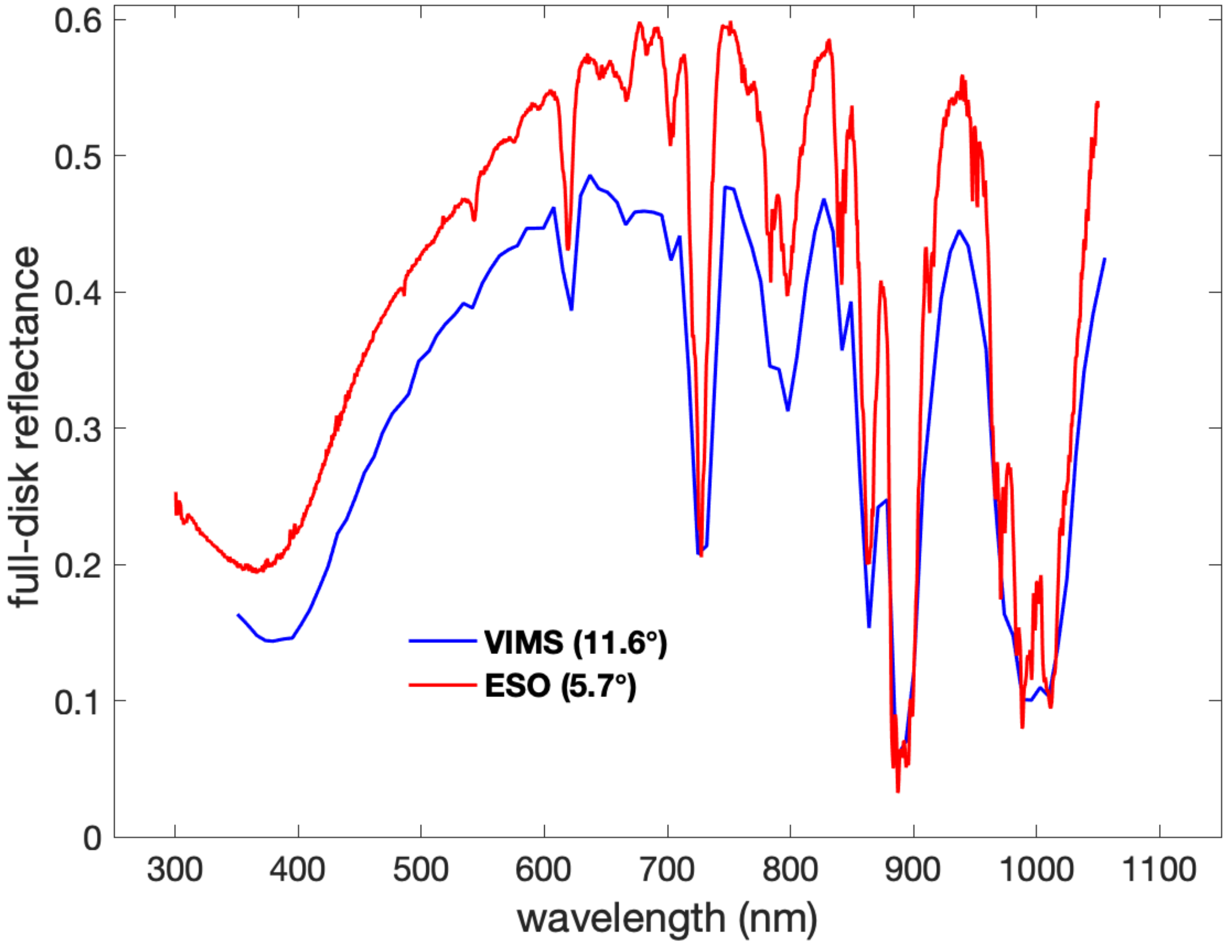

Figure S18. Comparison of Saturn's full-disk reflectance spectra between the European Southern Observatory (ESO) and the Cassini/VIMS observations. The VIMS and ESO data come from Fig. S16 and Fig S17, respectively.

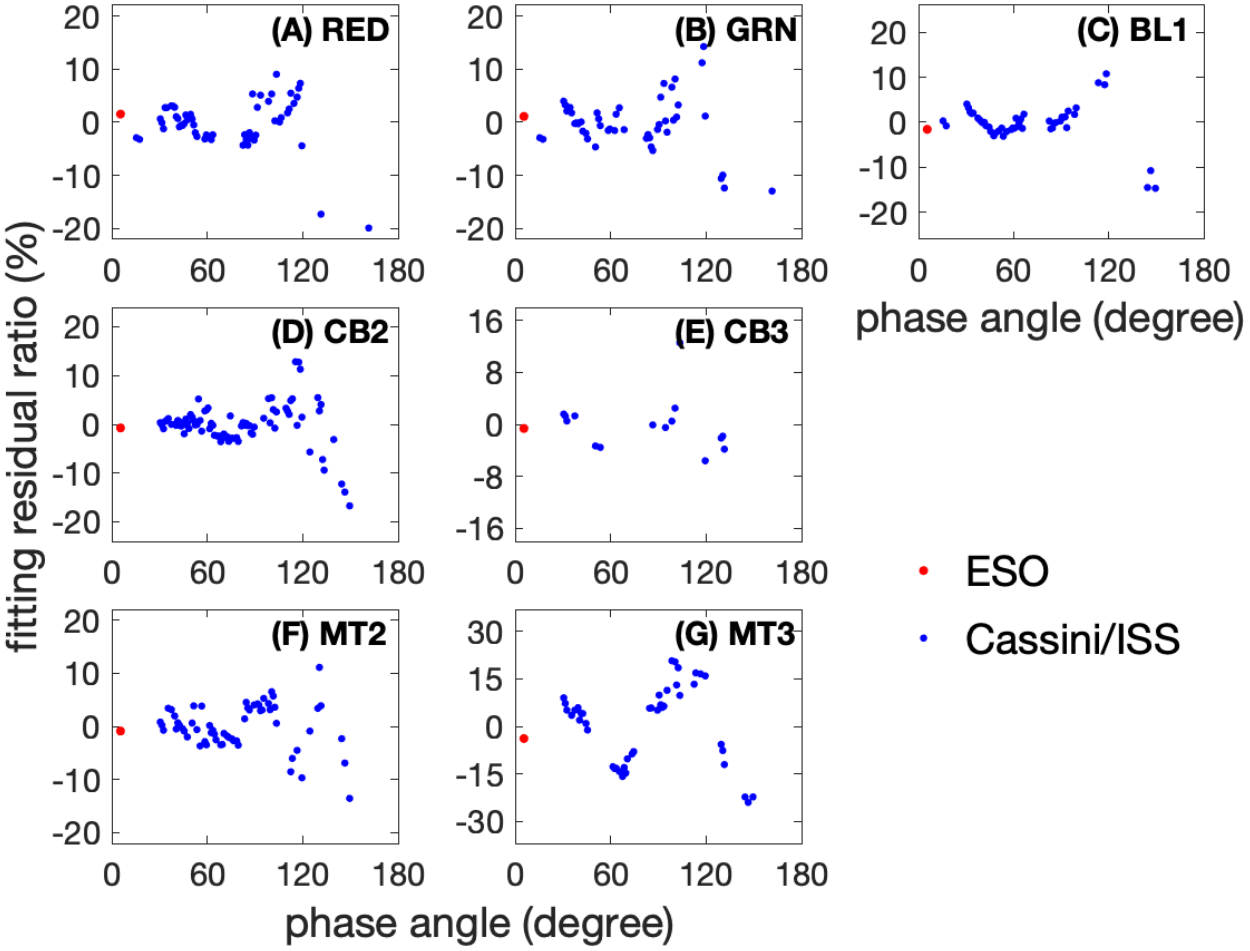

Figure S19. Ratios between fitting residual (fitting results – observational data) and observational data. The ratios are based on the fittings shown in Fig. 7 of the main text. (A) RED filter (647 nm); (B) GRN filter (568 nm); (C) BL1 filter (463 nm); (D) CB2 filter (752 nm); (E) CB3 fitler (939 nm); (F) MT2 filter (728 nm); and (G) MT3 fitler (890 nm).

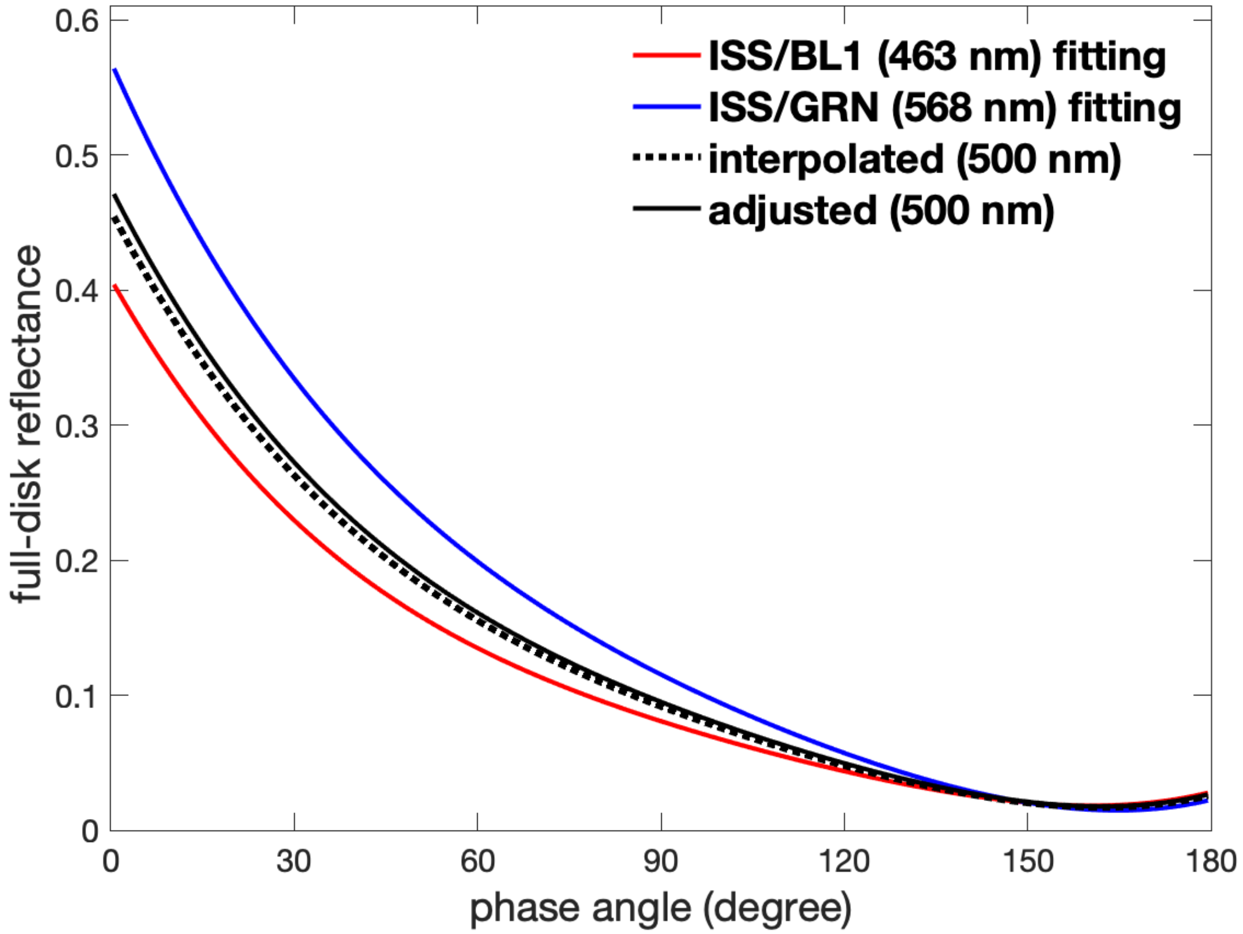

Figure S20. An example of filling the observational gaps in wavelength. We first linearly interpolate the complete phase functions at 463 nm (red line) and 568 nm (blue line) to the phase function at 500 nm (black dashed line). Then the interpolated phase function (black dashed line) is adjusted to the final phase function at 500 nm (black solid line) by the ESO observations (see Fig. S17). Please see discussion of this figure in Methods.

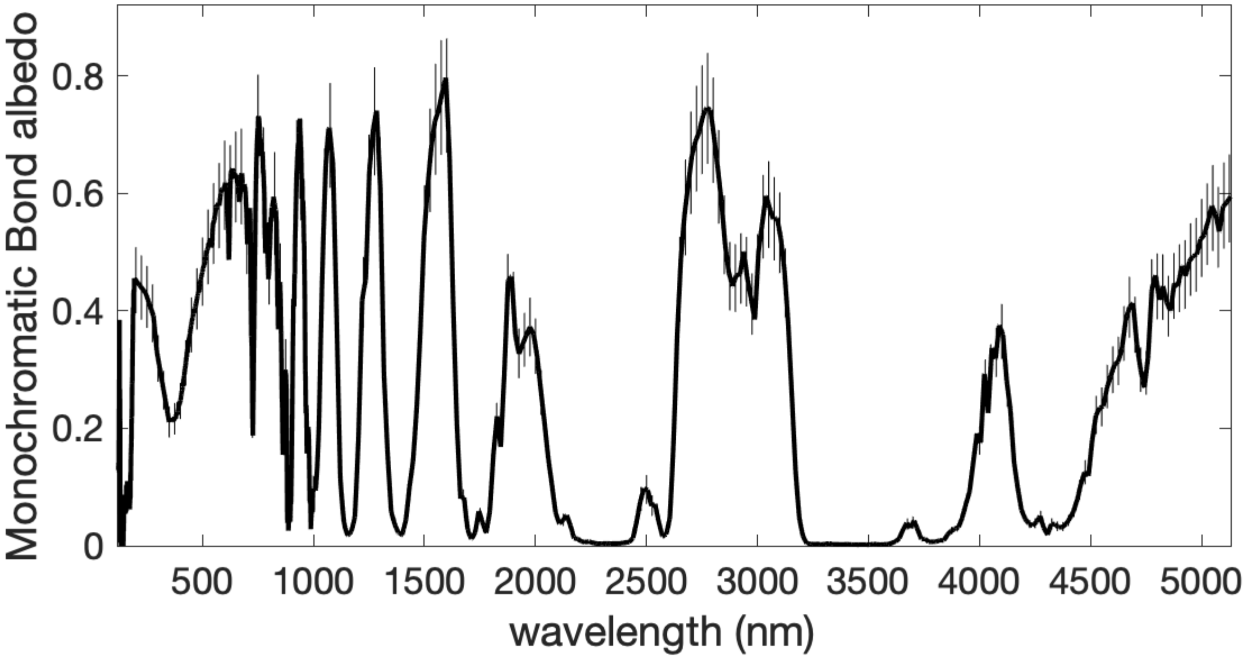

Figure S21. Saturn's monochromatic Bond albedo. The monochromatic Bond albedo is calculated by integrating the full-disk reflectance (Fig. 8 in the main text) across phase angles at each wavelength, following the methodology described in our previous studies[11,26].

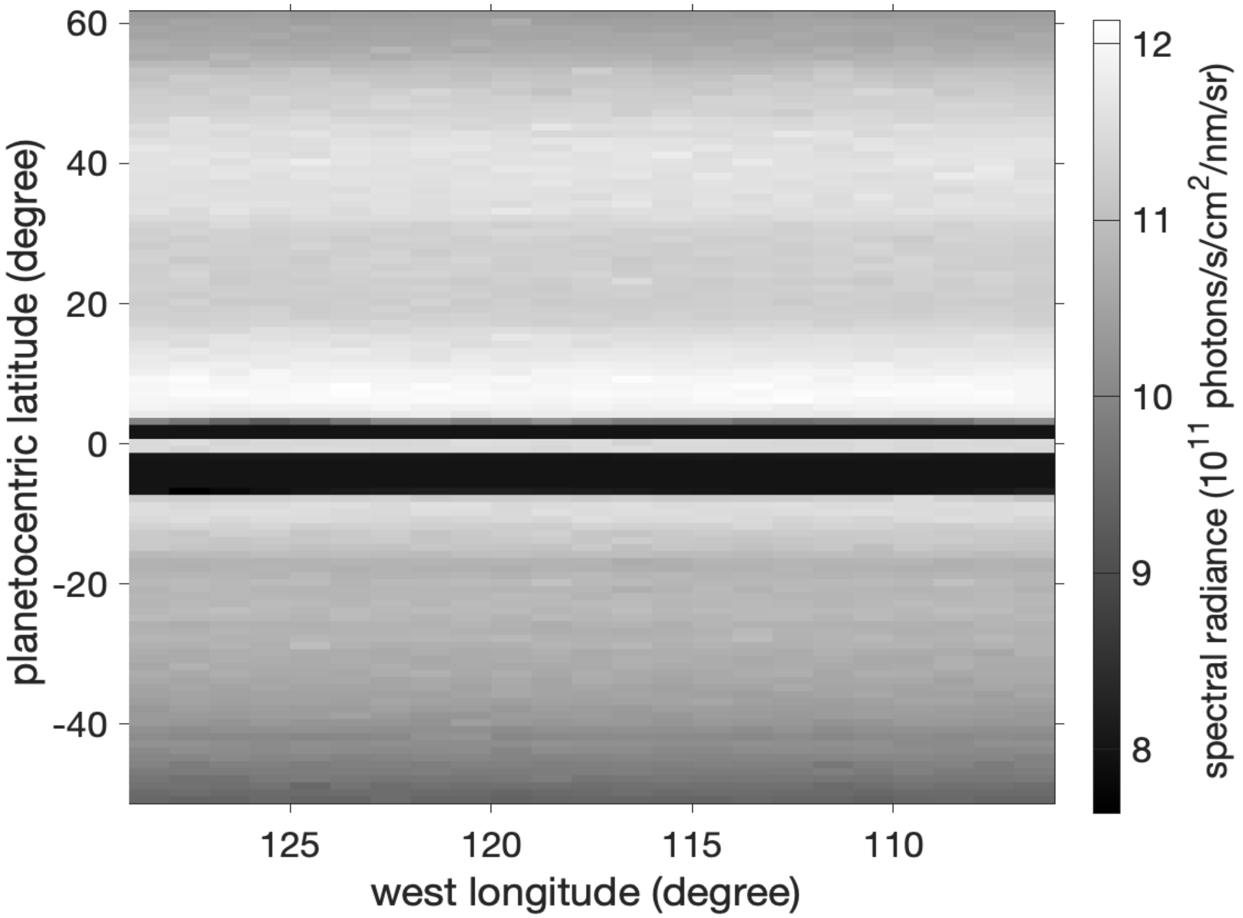

Figure S22. Banded structure of Saturn. The raw global image (image identification number W1657562957) was captured in July 2010 by the ISS BL1 filter (463 nm), with a phase angle of 87.5º and a spatial resolution of 140.4 km/pixel. Only a portion of the navigated global image is displayed here. At the time of observation, the sub-solar-latitude was 5.1ºN, causing the shadows cast by the rings to appear in the SH (i.e., the completely black latitude band just below the equator). The sub-Cassini-latitude was -1.9ºS, indicating that the Cassini view blocked by the rings is in the NH (i.e., the completely dark latitude band just above the equator). The bright latitude bands, which have relatively high reflectance, are referred to as zones. Conversely, the dark latitude bands, which have relatively low reflectance, are referred to as belts.

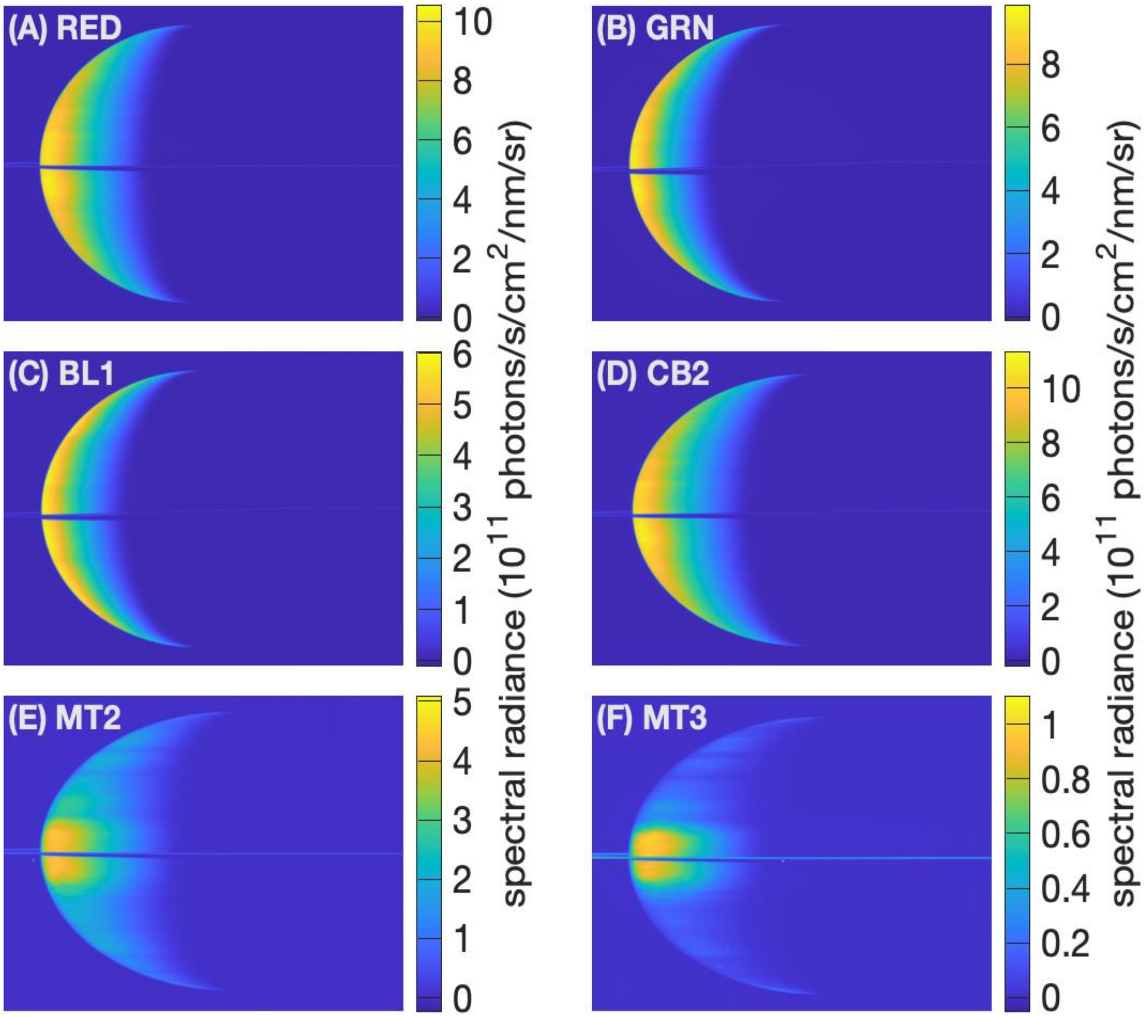

Figure S23. Examples of observations around the time when Saturn experienced the NH spring equinox (i.e., August 2009). The six raw images (image identification numbers are W1634877287, W1636880317, W1636880284, W1635000690, W1634819426, and W1634814263, respectively) were recorded by the ISS at six filters (RED at 647 nm, GRN at 568 nm, BL1 at 463 nm, CB2 at 752 nm, MT2 at 728 nm, and MT3 at 890 nm) respectively. These raw images were recorded between October and December 2009, with corresponding sub-solar latitudes ranging from 1.1°N to 1.8°N. The phase angles of these images changed from 103° to 119°, and the sub-Cassini-latitudes varied from 0.4°N to 0.5°N.

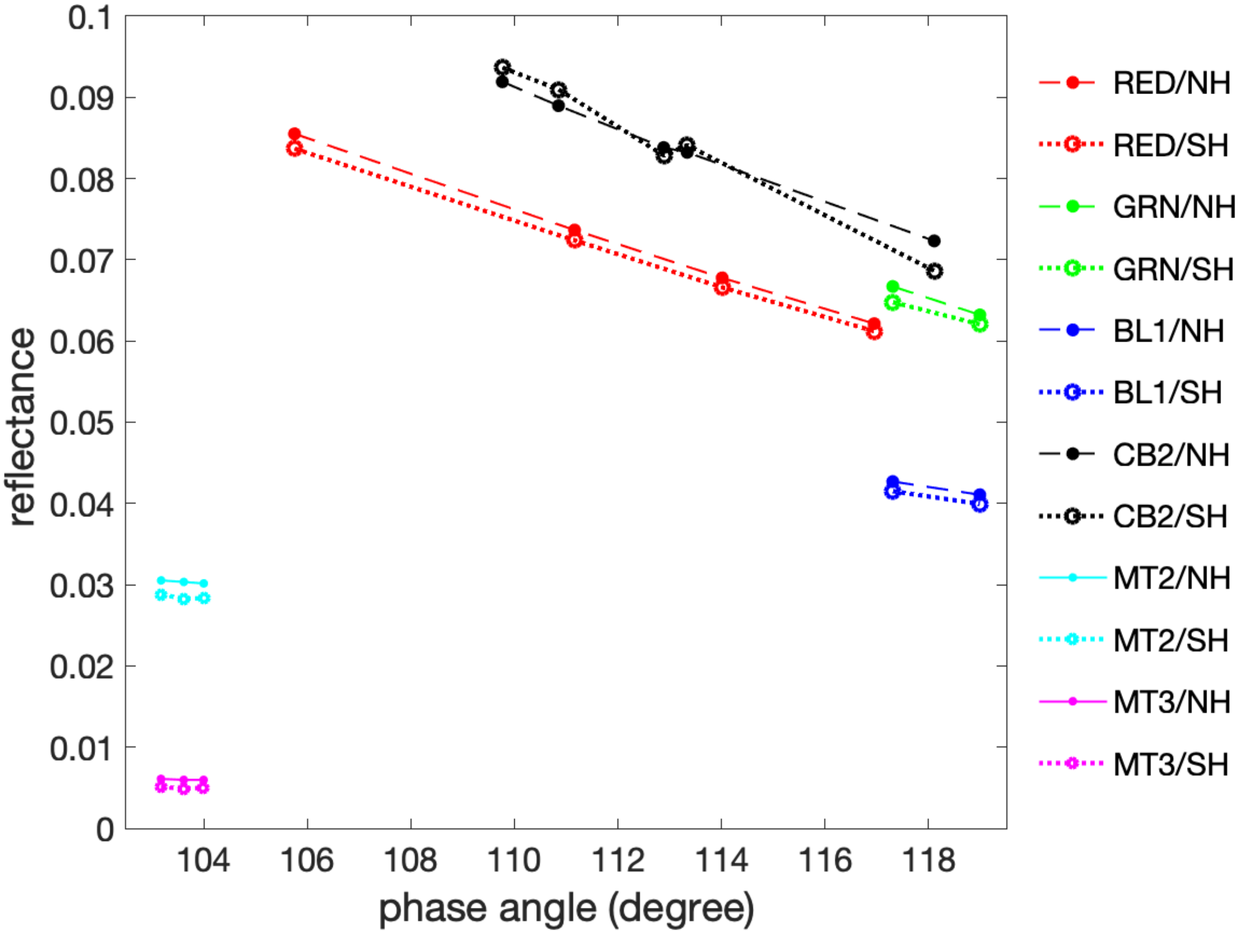

Figure S24. Hemispheric reflectance based on the ISS multi-filter global images recorded around the NH spring equinox. The calculation of hemispheric reflectance is based on these calibrated images shown in Fig. S23 (please also see Methods for more details).

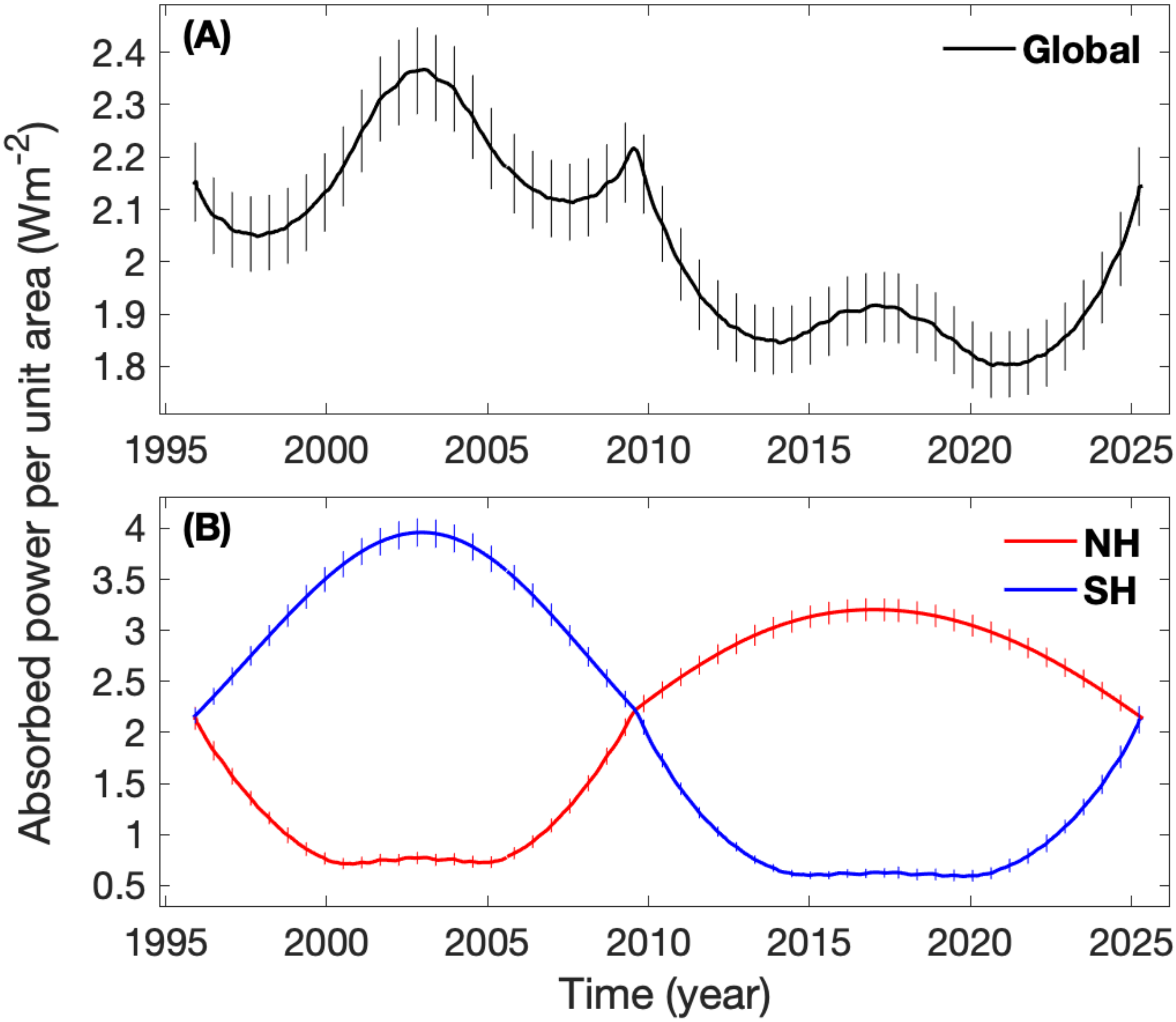

Figure S25. Saturn's global-average absorbed power for the Cassini epoch (2004-2017) and the complete orbital period from 1995 to 2025. The global and hemispheric absorbed powers are further compared to the corresponding emitted powers (see Figs 3 and 4 in the main text). The vertical lines in the two panels represent measurement uncertainties.
